# Rationale and study design of a cross sectional study documenting the prevalence of Heart Failure amongst the minority ethnic communities in the UK: the E-ECHOES Study (Ethnic - Echocardiographic Heart of England Screening Study)

**DOI:** 10.1186/1471-2261-9-47

**Published:** 2009-09-30

**Authors:** Paramjit S Gill, Russell Davis, Michael Davies, Nick Freemantle, Gregory YH Lip

**Affiliations:** 1 Primary Care Clinical Sciences, University of Birmingham, Edgbaston, Birmingham, B15 2TT, UK; 2 University Department of Medicine, City Hospital, Birmingham, B18 7QH, UK; 3 University Hospitals Birmingham NHS Foundation Trust, Edgbaston, Birmingham, B15 2TH, UK

## Abstract

**Background:**

Heart failure is an important cause of cardiovascular morbidity and mortality. Studies to date have not established the prevalence heart failure amongst the minority ethnic community in the UK. T'he aim of the E-ECHOES (Ethnic - Echocardiographic Heart of England Screening Study)is to establish, for the first time, the community prevalence and severity of left ventricular systolic dysfunction (LVSD) and heart failure amongst the South Asian and Black African-Caribbean ethnic groups in the UK.

**Methods/Design:**

This is a community based cross-sectional population survey of a sample of South Asian (i.e. those originating from India, Pakistan, Bangladesh) and Black African-Caribbean male and female subjects aged 45 years and over. Data collection undertaken using a standardised protocol comprising a questionnaire incorporating targeted clinical history taking, physical examination, and investigations with resting electrocardiography and echocardiography; and blood sampling with consent. This is the largest study on heart failure amongst these ethnic groups. Full data collection started in September 2006 and will be completed by August 2009.

**Discussion:**

The E-ECHOES study will enable the planning and delivery of clinically and cost-effective treatment of this common and debilitating condition within these communities. In addition it will increase knowledge of the aetiology and management of heart failure within minority ethnic communities.

## Background

Heart failure (HF) is an increasing cause of cardiovascular morbidity and mortality within the western world. It is important because it is common, costly, disabling and deadly. Further, it is also treatable. Nonetheless, the majority of data on the clinical epidemiology, prognosis and management strategies for heart failure have been derived predominantly from the white population. There is limited data on ethnicity and heart failure from the UK. [1-3]

Heart failure is a disabling condition, reducing self-reported quality of life more than most other chronic medical conditions. [4,5]; as well as being a major cause of healthcare expenditure. A recent estimate was that HF directly accounted for 1.9% of total NHS spending in the UK, with 69% of this being on hospitalisations, and indirectly (via long-term nursing care costs and secondary admissions) for a further equivalent of 2.0% of NHS expenditure. [6] Surveys in the UK and elsewhere show that 1-2% of the population as a whole and 10-20% of the very elderly have heart failure. [7,8] A large primary care based study in the West Midlands, the ECHOES study, found that prevalence of definite or probable heart failure in a predominantly White population aged 45 and older was 3.1%. [9] The increasing numbers of elderly will almost certainly mean there will be a further increase in the prevalence (and incidence) of heart failure over the next 20 years. [10] The symptoms and prognosis of patients with overt HF due to left ventricular systolic dysfunction (LVSD) are improved significantly by angiotensin converting enzyme inhibitors [11] and beta-blockers [12,13]; and ACE inhibitors in patients with asymptomatic LVSD can also delay or prevent progression to symptomatic heart failure [14,15]. Recent evidence suggests that different ethnic groups may respond differently to these therapies. [1,16] Finally, HF has a very high mortality, at around 80% in men within six years of diagnosis [17], a prognosis worse than most forms of cancer.

### Black minority ethnic groups in the United Kingdom

There were 4.6 million people (7.9%) from the Black and minority ethnic groups in the 2001 Census. The Black, Indian, Pakistani and Bangladeshi groups comprised 2%, 1.8%, 1.3%, 0.5% respectively. [18] The majority reside in large metropolitan areas and Birmingham has the largest proportion outside London.

Cardiovascular morbidity and mortality are substantially higher amongst these groups than the White population. [18] South Asians (SAs) living in the UK (i.e. those originating from India, Bangladesh or Pakistan), have a 50% greater risk of dying prematurely from coronary heart disease than the general population.[18] In contrast, premature death rates from CHD for Caribbeans and West Africans are much lower than average - around half the rate found in the general population for men and two-thirds of the rate found in women - despite the fact that hypertension is much commoner amongst these groups. Another major risk factor for HF is diabetes mellitus, which is much more common in African-Caribbean and South Asian minority groups than in the population as a whole.

Importantly, the difference in the death rates between SAs and the rest of the population is increasing. This is because the death rate from CHD is not falling as fast in SAs as it is in the rest of the population. From 1971 to 1991, the mortality rate for 20-69 year olds for the whole population fell by 29% for men and 17% for women, whereas in SAs it fell by 20% for men and 7% for women. [19] As myocardial ischaemia/infarction is the commonest cause of LVSD which is the most important and serious functional abnormality in patients with HF, it is to be expected that the prevalence of HF would be much higher in SAs.

### Heart failure in Black African-Caribbean and South Asians in the UK

The prevalence of HF in the SAs is currently not known as these groups have generally been underrepresented in previous studies. [1,9,20] Indeed, we are not aware of any population based epidemiological studies amongst these populations in the UK or other countries.

As is the case in African-Americans, HF occurs at an earlier age in SAs than in the White community. [21-26] Although no prospective data exist, a reanalysis of the data from our own study of acute HF admissions to a UK city centre hospital [21] has suggested that the relative risk of HF in those aged 60-79 years was 3.1 (95% CI 1.9-4.9) for African-Caribbean, and 5.2 (95% CI 3.7-7.4) for SAs. At 8 years' follow-up, the total mortality was 90.5% amongst whites and 87.0% amongst non-whites (Log Rank test, p = 0.07) where the non-white patients had numerically better survival at all time points until 6 years, after which the survival curves started to converge. [27] However, since African-Caribbean and SA patients were younger than their white counterparts, age at death appeared younger in these groups.

In addition to methodological issues with these studies conducted in secondary care, they have also focussed on particular ethnic groups and analysis have been reported by aggregating the groups, thereby masking the differences that may exist between the groups, particularly the SA category. [28,29] Indeed, we recognise that there are known differences in the subgroups (i.e. Indian, Pakistani, Bangladeshi) within the SA category. [30] Therefore, a primary care based study to determine the prevalence of LVSD and the syndrome of HF in this ethnic group is needed. Several of the investigators in this study were involved in the ECHOES study [9], in which the large majority of subjects studied were white.

## Primary Objective

To establish the community prevalence and severity of LVSD and heart failure [31] amongst the South Asian (Indian, Pakistani, Bangladeshi) and Black African-Caribbean ethnic groups in the UK.

## Methods/Design

### Ethical approval

The Walsall Local Research Ethics Committee has reviewed and approved the protocol (05/Q2708/45). Verbal and written consent will be obtained.

### Recruitment

This is a cross-sectional population survey of a sample of SA and Black African-Caribbean male and female residents of Birmingham aged 45 years and over. Recruitment entails a two-staged process with initial sample of general practices known to have high proportion of these minority ethnic patients and then a sample using the patient age-sex register.

Using 2001 Census data wards, serving the Birmingham Special Health Authority area, having >5% SAs and/or Black African-Caribbean as residents will be selected and general practices recruited from these wards.

**Figure 1:**
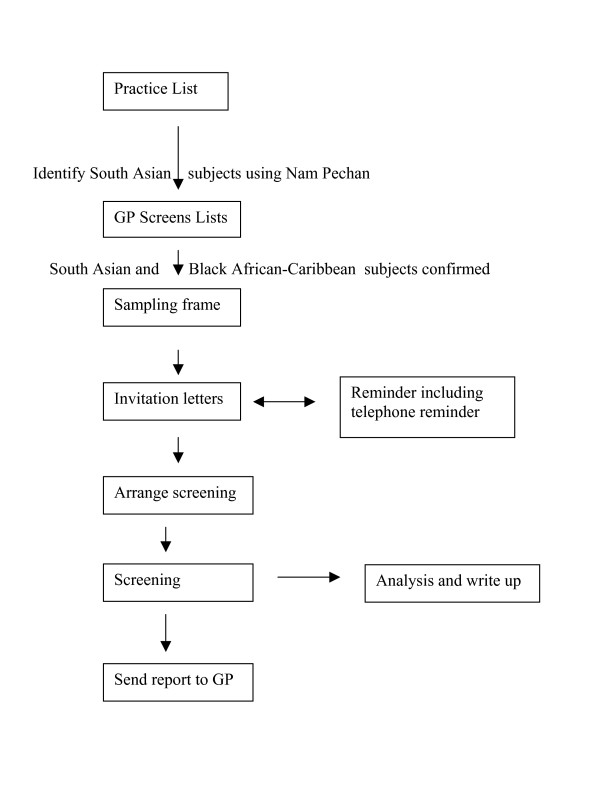
**Flow of participants through study**.

From the practice age-sex registers, all eligible subjects will be selected by sex and aged 45 years and over. As well as making the study directly comparable to ECHOES [9], it is also important to include those aged 45 and over in view of the earlier onset of ischaemic heart disease in SAs [18], which could result in a significant prevalence of HF in those aged 45-54 years. Subjects in the high-risk groups (i.e. hypertension, diabetes mellitus, ischaemic heart disease) will also be included, but not 'singled out' prospectively. Some of the general practices record ethnic group, but as ethnic group collection is not mandatory in primary care [32], we will use a combination of methods to identify the subjects. For SAs, we will screen the list for SA names using the Nam Pechan software that has been shown to be valid [33]; and for Black African-Caribbean subjects we will consult practice staff (see Figure 1). The general practitioner will then review the lists to ensure that only SA and Black African-Caribbean subjects are included and exclude any whom they consider it inappropriate to approach; for example, due to terminal illness or dementia. Finally, all eligible subjects will be asked their ethnic group before booking an appointment.

For pragmatic reasons, all eligible subjects will be invited for screening from each practice as minority ethnic groups tend to register with a specific practice in each area so that with random sampling, we would have to recruit not only a larger number of practices but these will also be dispersed over a number of health authority areas. This will lead to co-ordination and recruitment difficulties.

To facilitate recruitment, publicity in local media and consultation with community leaders will be undertaken, in addition to letters of invitation (in the appropriate languages) being sent to each potential subject inviting them to attend their practice. Further, we plan for interpreting provision and using the Health Survey for England 1999 [34] anticipate that 20% of the subjects will need interpreting due to poor spoken English.

### Baseline assessment of subjects

Subjects invited to attend for an assessment at their local general practice. At the clinic, the Research Fellow and Research Associate collected data by interview-administered questionnaire, undertook a physical examination, and performed an ECG and echocardiogram.

The Questionnaire includes the following data: age; date of birth; address; post code; self-determined ethnicity; religion; place of birth; migration history; languages spoken; level of education; alcohol consumption; cigarette smoking including other tobacco use; exercise assessment; history of illness in self and family; current medication; dyspnoea scoring leading to New York Heart Association functional classification All these measures are based on existing surveys such as the Health Survey for England [34] and the Fourth National Survey of Ethnic Minorities in Britain. [35] Information on co-morbidity (myocardial infarction, angina, hypertension, heart failure, stroke, diabetes) will be obtained. These will be validated by reviewing the general practitioner notes.

At physical examination, the following measurements will be undertaken using standard procedures: Height, weight, body mass index, resting pulse, systolic and diastolic blood pressure using an automated sphygmomanometer, and waist measurement. The height of the JVP will assessed; the heart auscultated for murmurs and added sounds, and the chest examined for signs of congestion and other abnormalities. Hepatomegaly, ascites and peripheral oedema will also be searched for. A resting 12 lead ECG (Mortara ELI 150) will be recorded, and coded independently by two cardiologists using the Minnesota criteria. [36]

Echocardiography will performed within the general practice surgeries by trained research fellows. This will be done using a portable VIVID i machine (GE Healthcare, Chalfont St Giles, UK), which produces good image quality and has tissue harmonic imaging capability and high quality colour and spectral Doppler facilities. Chamber dimensions will be obtained from the parasternal windows, and the presence and degree of left ventricular hypertrophy noted. Left ventricular function will be measured objectively using an area-length method from the apical four-chamber view. In cases where an objective measurement of left ventricular ejection fraction is not possible, a qualitative assessment will be made, that is, definite impairment (LVEF <40%), borderline (40-50%) and preserved (>50%), consistent with the investigators' normal clinical practice. Valve disease will be assessed semi-quantitatively and recorded, along with any other abnormalities. Parameters of diastolic function (mitral valve E:A ratio; E wave deceleration time; and isovolumic relaxation time) will also be measured. Tissue Doppler studies of the motion of the mitral valve annulus will also provide further insights into diastolic function.

HF will be defined using explicit criteria following the European Society of Cardiology (ESC) guidelines,[31] taking into account both symptomatic status and objective functional abnormalities on echocardiography.

A random blood sample will be obtained from those consenting by atraumatic venepuncture and stored at 4°C for up to 4 hours before transportation to the central laboratory for storage at -70°C for batch analysis. Initial analyses include renal function and lipids using routine automated methodology using reagents from Roche Diagnostics within a clinical Biochemistry laboratory (Lewes, UK). Similarly, HbA1c was measured using semi-automated HPLC methodology (Menarini, Berkshire, UK). As patients will be non-fasting, plasma triglyceride levels not measured. BNP measured using an established automated immunoassay technique using a commercially available assay (ADVIA Centaur, Bayer Healthcare, Newbury, UK). In addition, a full blood count will be tested. We will also store DNA at -70°C for future genetic analysis.

All subjects will be followed for long term outcomes.

### Quality control measures

The research team will be provided with training at the start of the project on administering the questionnaire; performing physical examination, the ECG and echocardiogram. The research team will follow written standard operating procedures that include a number of quality control checks, including re-reporting by a senior cardiologist of all abnormal echocardiograms and a sample of those reported by the research fellow as normal. As stated above, we will follow the same methodology as in the ECHOES study [9] and ensure between study quality control as 2 of the assessors (RCD, MKD) were also investigators in the ECHOES study.

### Feedback to informants

Each subject will be asked if they want their results to be forwarded to their GP. Written consent will be obtained for this. If any study results gave cause for concern, subjects will be asked to make an appointment with their GP.

## Sample Size and Statistical Analysis

### Response rate

It is stated that response to surveys by minority ethnic groups are low but this is not borne out by the extensive study by Bhopal et al [28] who achieved a 68% response rate. In the Health Survey for England [34], the response rate was over 80% in all six ethnic groups for interview, although this fell to below 50% for blood sampling. Our conservative estimate is a response of 50%, although every effort will be made to achieve a high response rate. In the predominantly white population studied in ECHOES [9], a 62.9% response rate was achieved by the investigators. Non-responders will be contacted by telephone where possible, and by a second letter of invitation.

### Principal Question - Prevalence of heart failure in SA population

The principal question addressed in this study is to estimate the prevalence of HF in the SA population. The precision of estimation of prevalence is dependent upon the number of subjects and the prevalence rate. Given the sample size of 3000 subjects, the 95% confidence intervals around a prevalence estimate of 2.3% will be ± 0.5%. Because of the properties of the binomial distribution, the width of confidence intervals will increase with increasing prevalence of heart failure within the plausible range of values (e.g. to 50% prevalence). Thus, with a prevalence of heart failure of 5%, the 95% confidence intervals will be ± 0.8%, and for 10% prevalence they will be ± 1.1%.

### Principal comparison - SA versus white group

This comparison will be undertaken making comparison with the rate of heart failure identified in the ECHOES study.[9] For this comparison, the study as planned will be have statistical power (1-β) of 80% to find a 1.3% difference in prevalence of heart failure as statistically significant at the conventional 2 sided α of .05. Similarly the study will have 90% power (1-β) of 90% to find a difference of 1.5% statistically significant.

Further we will describe the prevalence of heart failure in each of 3 SA population subgroups. In each subgroup, 1000 subjects will be recruited. The 95% confidence intervals around the estimate of prevalence for each subgroup would be ± 1.0% for a prevalence rate of 2.8%, ± 1.4% for a 5% prevalence, and ± 1.9% for a prevalence of 10%.

While our principal aims in this study are to provide reliable estimates of the prevalence of HF in the broad SA group and subgroups, we will also be in a position to compare explicitly the rates of heart failure in different groups. The statistical power for different comparisons depends upon the reference group rate, and this is described in the table below for different levels of statistical power (1-β) and for different comparator or reference group rates).

### Principal Question - Prevalence of heart failure in AC population

With a given 2% prevalence rate amongst the white population in ECHOES, we can make some direct contrast with that population. If we recruit 2000 patients in E-ECHOES AC, we have 90% power (1-beta) to find a 1.5% absolute difference in prevalence of heart failure between the two studies as statistically significant at the standard alpha level (two sided) of 5%. Further, within the proposed study, we have 90% power to find statistically significant differences of around 2.25%, given the range of comparator group sizes and of prevalence rates. Table 1

**Table 1:** Statistical power for different comparisons between subgroups

**Comparator Rate (n = 1000)**	**80% power (1-β) (n = 1000) to find the following difference significant at α = .05**	**90% power (1-β) (n = 1000) to find the following difference significant at α = .05**
3%	2.5%	3.0%

4%	2.8%	3.4%

5%	3.0%	3.6%

6%	3.3%	3.9%

7%	3.5%	4.1%

8%	3.7%	4.4%

9%	3.9%	4.6%

10%	4.1%	4.8%

### Analysis Plan

Descriptive analyses will be performed on all study variables, describing rates as percentages, and continuous variables as medians and lower and upper quartiles. Observed proportions of interest (notably heart failure prevalence estimates) will be described with 95% confidence intervals. For the comparisons of heart failure rates by survey, an exact test and confidence interval will be used to describe the estimated differences between the groups. In addition, a further exploratory multivariable binomial mixed analysis will be conducted using individual subject data from both studies, making appropriate adjustment for age, sex and practice (as a random effect).

## Discussion

The E-ECHOES study will be the largest community study documenting for the first time community prevalence of left ventricular systolic dysfunction and heart failure amongst the South Asian and Black African-Caribbean ethnic communities. It will provide pre-existing co-morbidity data; and follow-up will enable survival from heart failure amongst these communities.

It will provide the evidence base for the planning and management of this common disabling condition within the community.

## Competing interests

NF has received funding for research, consulting and speaking from a range of companies which manufacture treatments for heart failure or other cardiovascular therapies.

## Authors' contributions

PG and GL led the grant writing group. All authors were involved in the development and application of the protocol. The contributions of other members of the E-ECHOES study team are gratefully acknowledged and listed below. PG is the guarantor of this paper.

## Pre-publication history

The pre-publication history for this paper can be accessed here:


